# Ligand Binding Study of Human PEBP1/RKIP: Interaction with Nucleotides and Raf-1 Peptides Evidenced by NMR and Mass Spectrometry

**DOI:** 10.1371/journal.pone.0036187

**Published:** 2012-04-27

**Authors:** Laurette Tavel, Lucie Jaquillard, Andreas I. Karsisiotis, Fabienne Saab, Laurence Jouvensal, Alain Brans, Agnès F. Delmas, Françoise Schoentgen, Martine Cadene, Christian Damblon

**Affiliations:** 1 Department of Chemistry, University of Liège, Liège, Belgium; 2 CBM, CNRS, Orléans, France; 3 Institut de Chimie Organique et Analytique (ICOA), University of Orléans, CNRS FR 2708, UMR 7311, Orléans, France; 4 CIP, University of Liège, Liège, Belgium; 5 IMPMC, University Pierre & Marie Curie (P6), Paris, France; Institute of Enzymology of the Hungarian Academy of Science, Hungary

## Abstract

**Background:**

Human Phosphatidylethanolamine binding protein 1 (hPEBP1) also known as Raf kinase inhibitory protein (RKIP), affects various cellular processes, and is implicated in metastasis formation and Alzheimer's disease. Human PEBP1 has also been shown to inhibit the Raf/MEK/ERK pathway. Numerous reports concern various mammalian PEBP1 binding ligands. However, since PEBP1 proteins from many different species were investigated, drawing general conclusions regarding human PEBP1 binding properties is rather difficult. Moreover, the binding site of Raf-1 on hPEBP1 is still unknown.

**Methods/Findings:**

In the present study, we investigated human PEBP1 by NMR to determine the binding site of four different ligands: GTP, FMN, and one Raf-1 peptide in tri-phosphorylated and non-phosphorylated forms. The study was carried out by NMR in near physiological conditions, allowing for the identification of the binding site and the determination of the affinity constants K_D_ for different ligands. Native mass spectrometry was used as an alternative method for measuring K_D_ values.

**Conclusions/Significance:**

Our study demonstrates and/or confirms the binding of hPEBP1 to the four studied ligands. All of them bind to the same region centered on the conserved ligand-binding pocket of hPEBP1. Although the affinities for GTP and FMN decrease as pH, salt concentration and temperature increase from pH 6.5/NaCl 0 mM/20°C to pH 7.5/NaCl 100 mM/30°C, both ligands clearly do bind under conditions similar to what is found in cells regarding pH, salt concentration and temperature. In addition, our work confirms that residues in the vicinity of the pocket rather than those within the pocket seem to be required for interaction with Raf-1.

## Introduction

Phosphatidylethanolamine binding protein 1 (PEBP1), also known as Raf kinase inhibitory protein (RKIP), is involved in several processes in living cells. Its physiological function, mechanism of action and binding properties have been studied by using various cells and tissues from human, bovine, rat and mouse. The main results have revealed that PEBP1/RKIP regulates three key mammalian signaling pathways, namely Raf/MEK/ERK, NF-κB and G-protein coupled receptors (GPCR), and is implicated in signaling [1]–[3], proliferation [4], differentiation [5], migration [6], survival [7], and cell apoptosis [8], [9]. PEBP1 acts by direct interaction with the protein kinases involved in the pathways, such as Raf-1 [1], [10], MEK and ERK [11]. The interaction of PEBP1 with these protein kinases leads to their inhibition. As an example, the phosphorylation of Raf-1 by p21-activated kinase (PAK) and by Src family kinases, which is required for Raf-1 activity, is prevented by PEBP1 binding [12]. Bound Raf-1 is then inactive as a MEK kinase, which deregulates the ERK pathway. Upon phosphorylation by PKC on Ser153, PEBP1 dissociates from Raf-1 and inhibits the G-protein-coupled receptor kinase 2 (GRK2), which is a negative regulator of GPCRs [13], [14]. PEBP1 has also been shown to bind NF-κB inducing the kinase NIK and to inhibit the signaling mediated by NF-κB which plays a prominent role in apoptosis [2].

More specifically in human, hPEBP1 has been identified as a metastasis suppressor [15] since hPEBP1 expression is decreased in metastatic prostate [16], [17] and breast [18], [19] cancers. Moreover, hPEBP1 is a cell sensitizer to chemotherapy and immunotherapy [20]. Finally, hPEBP1 may also be involved in Alzheimer's disease [21], infertility [22], [23], and diabetes [24].

hPEBP1 is a member of the phosphatidylethanolamine binding protein (PEBP) family, which is a highly conserved group of more than 400 ubiquitous proteins found in a variety of tissues from a wide range of organisms (bacteria, yeasts, insects, mammals and plants). The crystal structures of PEBPs have revealed a remarkably conserved ligand-binding pocket. X-ray studies for bovine and human PEBP1s showed that ions such as acetate and o-phosphorylethanolamine (PE) (PDB 1A44; PDB 1B7A) [25], phosphate and o-phosphotyrosine (PDB 2QYQ) [26], or cacodylate (PDB 1BEH) [27] could bind to this conserved pocket. The conserved pocket is the only ligand-binding site of PEBP1s identified by X-ray.

Besides crystallographic data, binding studies have been reported using other techniques. A study by affinity chromatography at pH 7.5 revealed that nucleotides could bind to the bovine brain PEBP (bPEBP), in the decreasing affinity order FMN>GTP>GDP>GMP>FAD>ATP>NADP>CTP>UTP>ADP [28]. Interactions of human and bovine PEBP1s with morphine and morphine derivatives were characterized at pH 6.8 by noncovalent mass spectrometry [29]. Moreover, an NMR study of rat PEBP1 (rPEBP1) in near physiological conditions (pH, salt concentration, temperature) showed that the conserved pocket could accommodate various ligands such as 1,2-dihexanoyl-sn-glycero-3-phosphoethanolamine (DHPE), dihexanoylphosphatidylserine (DHPS), dihexanoylphosphatidylglycerol (DHPG), and dihexanoylphosphatidic acid (DHPA) [30]. The screening of a chemical library by NMR spectroscopy revealed three novel ligands for rPEBP1 that also bind to the protein pocket [31]. Shemon and co-workers (2009) were also interested in the interaction of rPEBP1 with locostatin ((S)-(+)-4-benzyl-3-crotonyl-2-oxazolidinone), since it is known to be a cell migration inhibitor whose cellular target is PEBP1 in cell lines from different origins [6]. However, locostatin itself could not be analyzed by NMR because of its limited solubility and the fact that it induced protein precipitation [32]. Contrary to locostatin, its precursor (S)-4-benzyl-2-oxazolidinone was compatible with NMR studies, which indicated a binding to the conserved pocket of rPEBP1 [32]. Furthermore, interactions between rat, mouse or human PEBPs and an inhibitor of phosphodiesterase-5 (PDE5) were shown by combining affinity based enrichment and mass spectrometry [33]. The binding was confirmed by solution based assays using absorbance, fluorescence and NMR spectroscopy.

However, some of these studies have emphasized the importance of both experimental conditions and the species of the PEBP used in the binding studies. A comparative NMR study at pH 7.4 and 6.0 showed that some ligands of hPEBP1 and bPEBP1 previously identified did not interact with rPEBP1 at pH 7.4, particularly PE [30] and the nucleotides GDP and GTP [31]. Furthermore, the binding study involving PEBPs from rat, mouse and human (rPEBP2, mPEBP1, mPEBP2 and hPEBP1) and an inhibitor of PDE5 evidenced different behaviors depending on the species and the tissues of origin of the protein, in spite of high sequence homologies and high similarities in the protein tertiary structures [33].

As previously mentioned, PEBP1 from bovine, human or rat is able to bind small ligands as well as proteins such as the Raf-1, MEK and ERK kinases [1], [11]. Although the mechanism of PEBP1 binding to Raf-1 remains unknown, several studies have provided information about the binding region of Raf-1 on the one hand, and the binding region of PEBP1 on the other hand. Yeung and co-workers (2000) showed that the binding domains of Raf-1 with rPEBP1 were subdomains I and II, a region of approximately 100 amino acids [10]. More recent studies revealed that the phosphorylated N-region of Raf-1, encompassing amino acids 331 to 349, was sufficient to bind to rPEBP1 [34], [35]. These data are consistent with rat and human PEBP1s inhibiting Raf-1 by preventing its phosphorylation at S338 and Y341 [12]. Besides, it has been shown that binding to Raf-1 requires the integrity of the rPEBP1 pocket [30], [35] and is influenced by rPEBP1 pocket occupancy by another ligand (DHPE) [30]. Furthermore, the P74L mutation of the rPEBP1 pocket affects Raf-1 binding, but not the binding of DHPE to rPEBP1 [30]. Thus, the rPEBP1 lipid binding site may be distinct from the kinase binding site, and at least some of the pocket residues may be involved directly or indirectly in the interaction between rPEBP1 and Raf-1 [31]. Another work did support the idea of an indirect binding of Raf-1 to the PEBP1 pocket. Indeed, in contrast to DHPE, the locostatin precursor binding to the rPEBP1 pocket was not sufficient to interfere with Raf-1 binding [32]. The authors suggested that other residues of rPEBP1 may be critical for Raf-1 binding.

Thus, in spite of the numerous papers concerning PEBP1 binding ligands, one another's conclusions are not always in agreement. The works previously mentioned evidenced different binding behaviors as a function of (i) the species of PEBP1 (mouse, rat or human) [33], and (ii) the experimental conditions of binding, particularly the pH value [31]. Moreover, the binding of Raf-1 is complex and the binding site on PEBP1 is still unknown. In the present study, we investigated the human PEBP1 by NMR to determine the binding site of four different molecules: two nucleotides, GTP and FMN, because of their relatively high affinities for bPEBP1 [28], and a Raf-1 peptide of 19 amino acids in tri-phosphorylated and non-phosphorylated forms. The non-phosphorylated peptide RPRGQRDSSYYWEIEASEV is the minimal region 331–349 of Raf-1 required for rPEBP1 binding [34]. Three phospho-amino acids were incorporated at the positions Ser^338/339^ and Tyr^341^, since the phosphorylation enhanced the binding to rPEBP1 as studied by surface plasmon resonance [34]. In order to examine the effects of experimental conditions such as pH, salt concentration, and temperature on binding, we investigated hPEBP1 in two sets of conditions: MES 10 mM pH 6.5 at 20°C, and HEPES 10 mM, NaCl 100 mM, pH 7.5 at 30°C (near physiological conditions). NMR titrations were also used to derive the affinity constants K_D_ of the ligands with hPEBP1. Native mass spectrometry (MS) was used as an alternative method for measuring K_D_ at pH 7.4/37°C for GTP and FMN and at pH 7.4/25°C or pH 6.6/20°C for the tri-phosphorylated Raf-1 peptide.

## Results

^15^N-^1^H heteronuclear single quantum coherence (HSQC) NMR experiment was used to study the interaction between hPEBP1 and four different ligands under two sets of experimental conditions. The HSQC spectrum of a protein monitors peptidic NH groups, giving one signal per amino acid at the level of the protein backbone. Since the chemical shift is very sensitive to the environment of the observed nuclei, the binding of the ligand affects the chemical shifts of both peptidic nitrogen and proton within the binding area. Hence, the residues involved in a binding can be determined using HSQC spectra of hPEBP1 in the presence or absence of a ligand.

**Figure 1:**
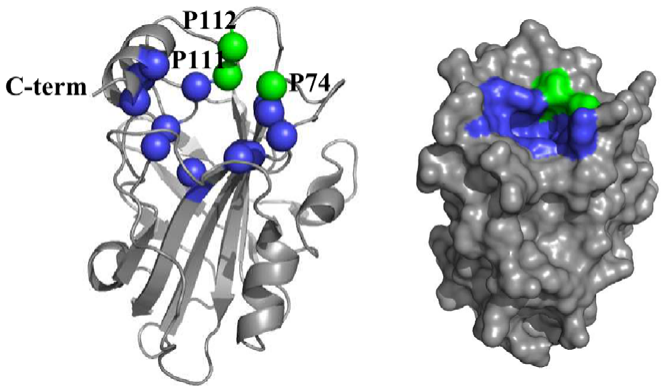
The hPEBP1 pocket on the X-ray ribbon structure and on surface representation (PDB 2QYQ). Residues indicated in blue are D70, A73, Y81, W84, H86, V107, G108, G110, H118, Y120, L180, Y181, and L184. Prolines 74, 111 and 112, which belong to the hPEBP1 pocket but are not detected by HSQC spectrum, are indicated in green.

Mammalian PEBP1s crystal structures (PDB 2QYQ [26]) have revealed a remarkably conserved ligand-binding pocket. The hPEBP1 pocket can be defined by 16 residues at the surface of the protein: D70, A73, P74, Y81, W84, H86, V107, G108, G110, P111, P112, H118, Y120, L180, Y181, and L184 (Figure 1).

### GTP and FMN do bind to the ligand-binding pocket of hPEBP1

**Figure 2:**
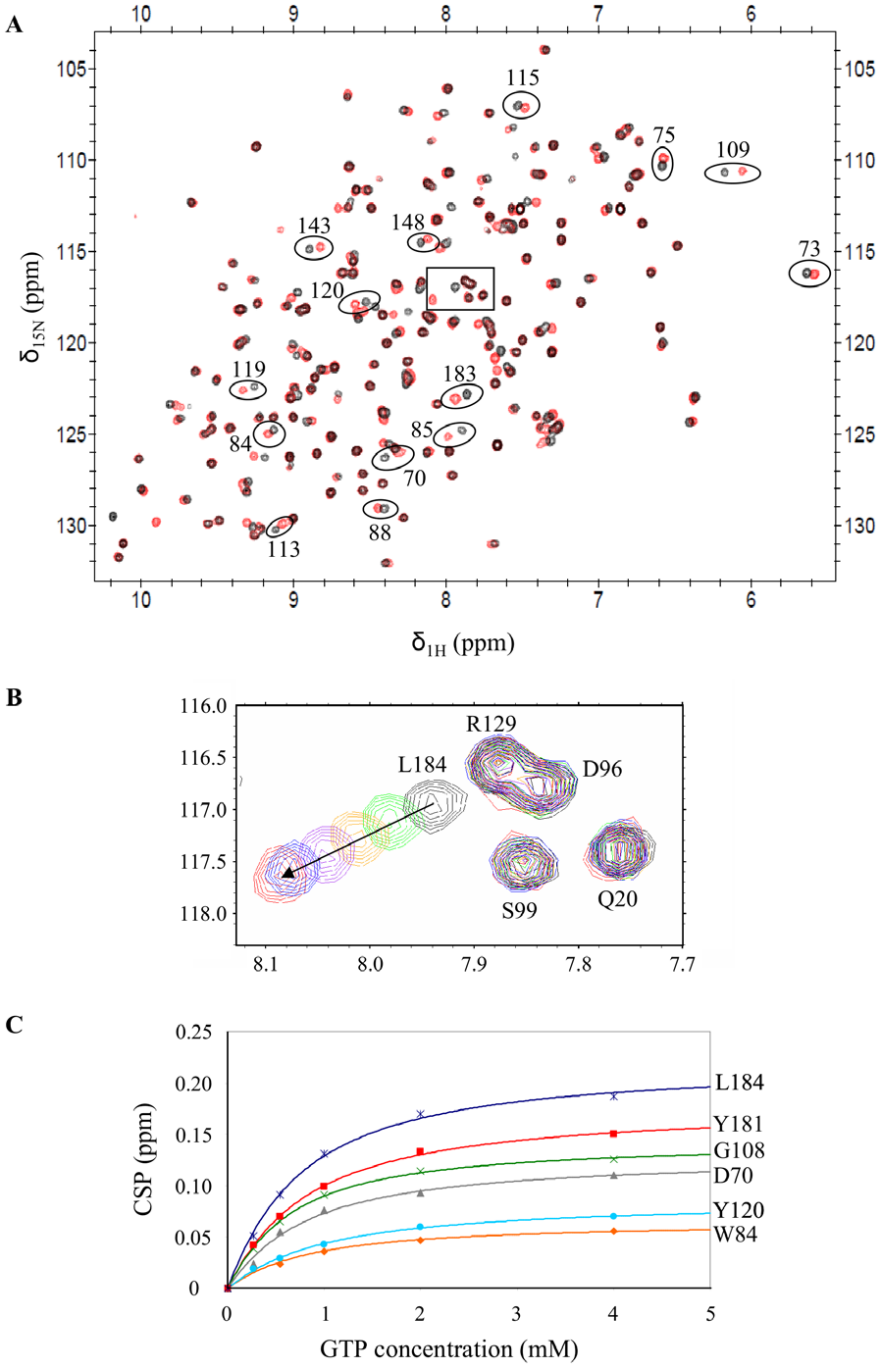
Binding of GTP to hPEBP1 at pH 6.5/20°C by NMR. (**A**) Overlay of ^1^H, ^15^N HSQC spectra of hPEBP1 270 µM in the absence (black) and presence (red) of GTP 4 mM. (**B**) Expansion of the selected HSQC region. Overlay of six HSQC spectra of hPEBP1 270 µM with increasing concentration of GTP: 0 mM (black), 0.27 mM (green), 0.54 mM (orange), 1 mM (purple), 2 mM (blue), and 4 mM (red). (**C**) Plot of CSP versus GTP concentration; data fitted against equation of K_D_ (see M&M) for the 6 residues indicated on Figure 3A.

**Figure 3:**
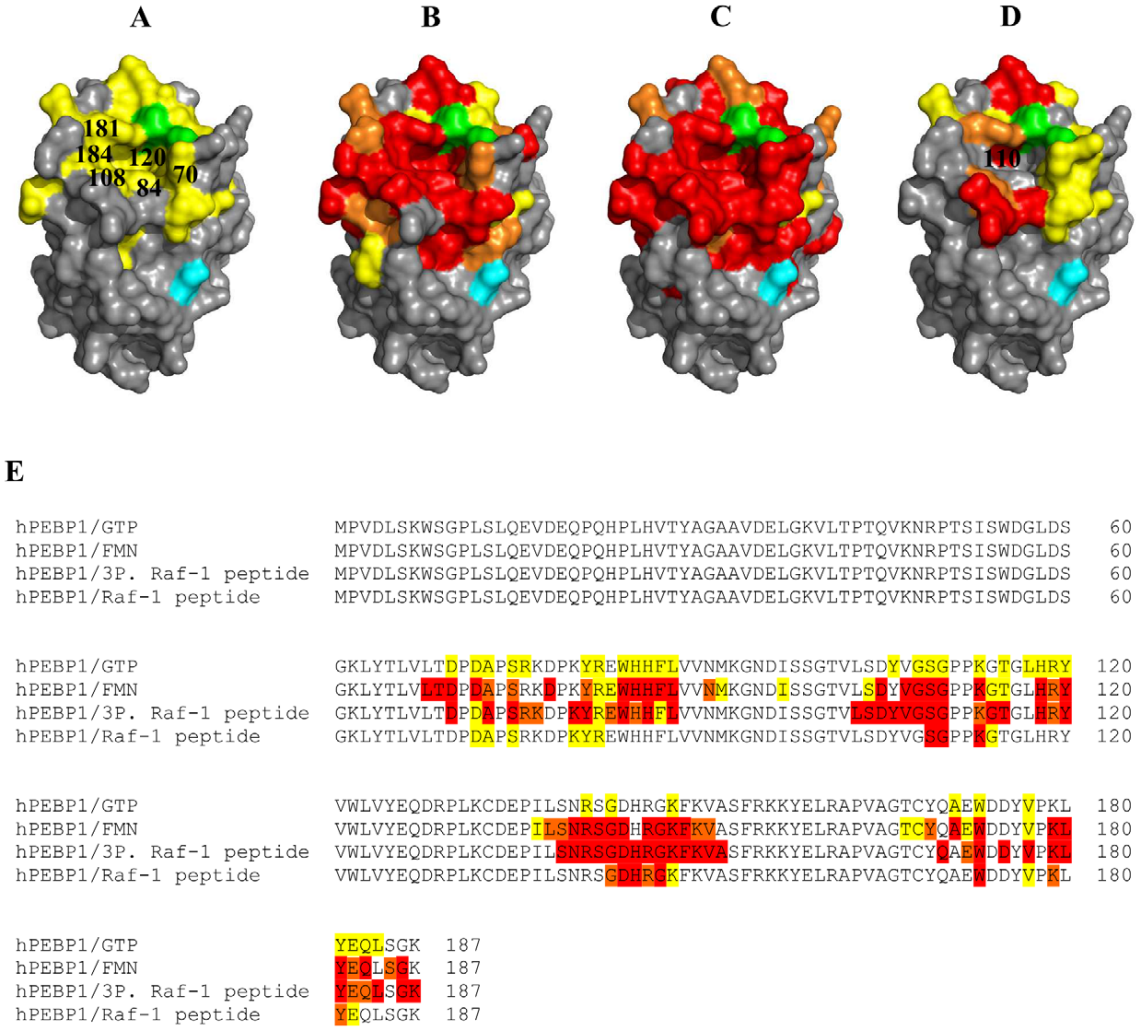
Binding site of ligands at hPEBP1 surface at pH 6.5/20°C. Mapping of amino acid residues whose HSQC peak is significantly affected by (**A**) GTP, (**B**) FMN, (**C**) the tri-phosphorylated Raf-1 peptide, and (**D**) the non-phosphorylated Raf-1 peptide at the surface of hPEBP1 (X-Ray; PDB 2QYQ). Red = residues in slow exchange; orange = residues in intermediate exchange; yellow = residues in fast exchange. Prolines 74, 111 and 112, which belong to the hPEBP1 pocket but are not detected by HSQC spectrum, are indicated in green. Serine 153 is indicated in cyan as a reference point. (**E**) hPEBP1 sequence alignment (accession number P30086) indicating the residues defining the binding surface of GTP, FMN, the tri-phosphorylated Raf-1 peptide (3P. Raf-1 peptide), and the non-phosphorylated Raf-1 peptide (Raf-1 peptide). The color code is similar to (D).

NMR titration of GTP in MES 10 mM pH 6.5 at 20°C revealed 34 residues in fast exchange on the NMR time scale (Figure 2A). Upon titration, the signals of these residues are shifted in both dimensions in the HSQC 2D plane, and the trajectories of the signals are linear, as observed for L184 (Figure 2B). This linear evolution indicates a single binding event [36]. Mapping of the residues affected by GTP binding on hPEBP1 structure corresponds to the conserved pocket of hPEBP1 (Figure 3A).

The chemical shift perturbations (CSP) values of the 34 perturbed residues were plotted versus the GTP concentration, and the data were fitted against equation 2 [37], giving a binding constant for each residue. Fitted titration data are shown in Figure 2C for six residues. CSP data were normalized to CSP_max_ (with the CSP_max_ estimate obtained from the curve fitting method used to calculate K_D_). The plot of normalized CSP (CSP/CSP_max_) versus the GTP concentration revealed a uniform behavior for 30 of the 34 perturbed residues (data not shown). The data of four residues (V27, G57, Y106, and G110) gave very different values of K_D_ compared to those calculated from the 30 other residues perturbed by GTP, and hence, were not considered for the estimation of the average binding constant of GTP. Among the four residues excluded for the K_D_ estimation of GTP, (i) two of them (V27 and G57) were isolated on the protein surface, (ii) one (Y106) belongs to the binding surface, but is far from the center of the hPEBP1 pocket, and (iii) the last one's peak intensity (G110) was too low to get data of quality. Thus, estimated from 30 perturbed residues, the average binding constant for GTP at pH 6.5/20°C is K_D_ = 669±140 µM (Table 1).

**Table 1:** K_D_ values of nucleotides derived from NMR and MS spectrometry.

Compound	NMR K_D_ (µM) pH 6.5/20°C	NMR K_D_ (µM) pH 7.5/NaCl 100 mM/30°C	MS K_D_ (µM) pH 7.4/37°C
GTP	669±140	3425±1967	89±48
FMN	14±9	252±84	5±4

A total of 67 residues were affected upon FMN titration in MES 10 mM pH 6.5 at 20°C. Most of these residues were in slow exchange on the NMR time scale (39 residues in red on Figure 3B) and defined a binding surface centered on the conserved hPEBP1 pocket. 15 residues in fast exchange and 13 residues in intermediate exchange on the NMR time scale were also observed (residues in fast exchange in yellow, and residues in intermediate exchange in orange on Figure 3B). These residues are located in the outermost region of the binding surface. Since slow exchange usually indicates a higher affinity compared to fast or intermediate exchange, the data show that the pocket corresponds to the region with the greatest affinity for FMN. However, the binding constant could not be calculated from the intensity data of the residues in slow exchange because their peak intensities dropped sharply when the FMN concentration increased. Hence, the affinity was estimated from CSP data of residues in fast exchange: K_D_ = 14±9 µM. This affinity is therefore underestimated.

### GTP and FMN do bind to hPEBP1 in near physiological conditions

Since the experimental conditions can affect the binding behavior [31], the binding of GTP and FMN was also investigated under near physiological conditions: HEPES 10 mM pH 7.5, NaCl 100 mM, at 30°C.

**Figure 4:**
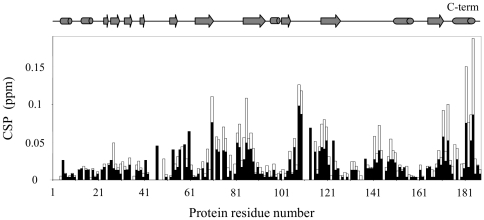
Comparison of GTP binding to hPEBP1 in two conditions. hPEBP1 chemical shift perturbations at GTP saturation concentration for both tested conditions: GTP 4 mM at pH 6.5/20°C (white), and GTP 6.4 mM at pH 7.5/NaCl 100 mM/30°C (black). CSP values are higher at pH 6.5/20°C than pH 7.5/NaCl 100 mM/30°C (hPEBP1 270 and 100 µM, respectively).

The binding of GTP in near physiological conditions exhibited the same features as in MES 10 mM pH 6.5, 20°C, that is, the same binding site and a fast exchange on the NMR time scale. Among the 34 residues affected at pH6.5/20°C, 26 were also perturbed at pH 7.5/NaCl 100 mM/30°C. However, the chemical shift perturbations were smaller in near physiological conditions (<CSP>+2σ = 0.058 ppm) than at pH 6.5/20°C (<CSP>+2σ = 0.087 ppm) (Figure 4). Moreover, the binding constant measured for GTP at pH 7.5/NaCl 100 mM/30°C was 3425±1967 µM, which was higher than 669±140 µM at pH 6.5/20°C (Table 1).

Similarly to the study performed at pH 6.5/20°C, the titration of FMN in near physiological conditions revealed residues in slow exchange on the NMR time scale, as well as residues in intermediate and fast exchange. The data evidenced the conserved hPEBP1 pocket as the binding surface in both conditions. Nevertheless, regarding the residues in slow exchange, the loss in intensity occurred at a higher FMN concentration at pH 7.5/NaCl 100 mM/30°C than at pH 6.5/20°C (data not shown). The estimation of K_D_ from CSP data of residues in fast exchange confirmed a lower affinity in near physiological conditions: K_D_ = 252±84 µM at pH 7.5/NaCl 100 mM/30°C versus K_D_ = 14±9 µM at pH 6.5/20°C (Table 1).

The formation of hPEBP1-nucleotide complexes was also monitored by native MS (data not shown). In ammonium bicarbonate (ABC) 20 mM at pH 7.4 and 37°C, hPEBP1 was found to bind with GTP and FMN with K_D_ values of 89±48 µM and 5±4 µM, respectively (Table 1). As in NMR, hPEBP1 showed a higher affinity for FMN than for GTP. In native MS, K_D_ values were measured in the absence of NaCl. In contrast, NMR measurements were performed in the presence of NaCl 100 mM, leading to a partial screening of electrostatic charges, and consequently to higher K_D_ values.

### The Raf-1 peptide does bind in tri-phosphorylated and non-phosphorylated forms to the ligand-binding pocket of hPEBP1

HSQC spectra displayed a total of 73 perturbed residues upon titration of the tri-phosphorylated Raf-1 peptide in MES 10 mM pH 6.5 at 20°C: 54 residues in slow exchange, 11 residues in intermediate exchange, and 8 residues in fast exchange (Figure 3C). Among these 73 perturbed residues, 11 residues were buried (V27, V67, L68, T69, D72, S109, V121, W122, V124, V151, and C168) and three were isolated at the surface of hPEBP1 (Q15, G57, and L58). Thus, after discrimination, we determined a single binding surface composed of 59 residues including and surrounding the conserved pocket. Neither the intensity data of the residues in slow exchange, nor the CSP data of the residues in fast exchange did allow us to estimate the affinity of the tri-phosphorylated Raf-1 peptide. Indeed, on the one hand, the peak intensities dropped sharply when the Raf-1 peptide concentration increased. And, on the other hand, the plot of CSP versus Raf-1 peptide concentration revealed no saturation upon titration.

**Figure 5:**
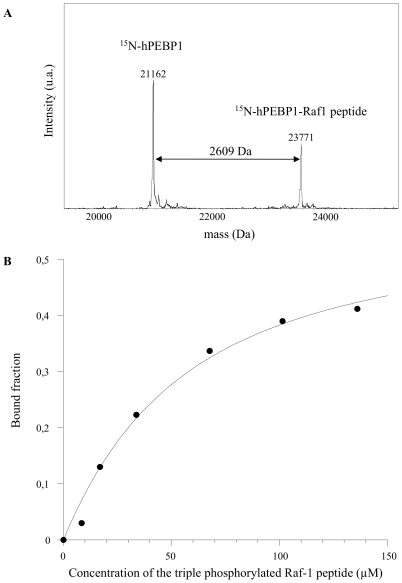
Binding of the tri-phosphorylated Raf-1 peptide to hPEBP1 by Mass Spectrometry. (**A**) ESI mass spectrum of hPEBP1 in complex with the tri-phosphorylated Raf-1 peptide, deconvoluted from 10+, 9+ and 8+ charge states. The complex was formed by incubating 18 µM hPEBP1 with 67.6 µM Raf-1 peptide at 20°C in 20 mM NH_4_OAc, pH 6.6. (**B**) MS-measured hPEBP1 bound fraction as a function of the tri-phosphorylated Raf-1 peptide concentration.

However, the affinity of hPEBP1 for the tri-phosphorylated Raf-1 peptide was measured by native MS. A K_D_ value of 45±12 µM was obtained in conditions of incubation similar to the conditions used for NMR, in ammonium acetate at pH 6.6 and 20°C (Figure 5). In ABC at pH 7.4 and 25°C, a K_D_ of 11±3 µM was found, a value close to the K_D_ of 20 µM determined in solution with rPEBP1 [34]. All these values are within the same order of magnitude.

For the NMR titration with the non-phosphorylated Raf-1 peptide in MES 10 mM pH 6.5 at 20°C, only 33 residues were perturbed: 16 residues in slow exchange, 6 residues in intermediate exchange, and 11 residues in fast exchange (Figure 3D). After discrimination of the buried residues (V27, V46, D72, and S109) and those isolated at the surface (Y29), three surface patches were identified. Two small surfaces were formed by L25-H26-V34-G166 and W55/D56/G57/L58/V164 on the opposite side of the conserved pocket of hPEBP1, but were not large enough to be considered as potential binding surfaces. Besides, 19 perturbed residues defined a surface centered on the conserved pocket similarly to the other ligands. It is worth noticing that the corresponding binding surface was larger for the tri-phosphorylated peptide. In addition, comparison of the peak intensities for both Raf-1 peptides titrations at similar protein/ligand ratios revealed that the intensity loss was less severe for the non-phosphorylated peptide (data not shown). Altogether, these data suggest a lower affinity of hPEBP1 for the non phosphorylated Raf-1 peptide compared to the tri-phosphorylated Raf-1 peptide, in agreement with literature reports [34]. However, we could not confirm this with a binding constant value. The severe drop of peak intensities for the residues in slow exchange and the non-saturation of CSP for the residues in fast exchange upon titration did not allow us to estimate the K_D_ as mentioned for the tri-phosphorylated Raf-1 peptide.

One additional difference could be observed between the two peptides regarding the perturbation of the pocket itself. The conserved pocket of hPEBP1 is formed by 16 residues at the surface of the structure PDB 2QYQ [26]. Three prolines are among these 16 residues, hence only 13 residues of the pocket can be detected by HSQC. Whereas all these 13 residues were perturbed upon binding of the tri-phosphorylated Raf-1 peptide, only four of them were affected by the binding of the non-phosphorylated peptide: three residues (A73, Y81, and G110) were located on the edge of the pocket, and only G110 was in the bottom of the pocket.

## Discussion

The present study demonstrates and/or confirms the binding of hPEBP1 to four ligands, two nucleotides and one Raf-1 peptide in tri-phosphorylated and non-phosphorylated forms. Although the affinities for GTP and FMN decrease as pH, salt concentration, and temperature increased from pH 6.5/NaCl 0 mM/20°C to pH 7.5/NaCl 100 mM/30°C according to our NMR data, both ligands clearly do bind under near physiological conditions. Moreover, all four ligands bind to the same region centered on the conserved pocket previously identified by X-ray crystallography.

### The binding of the two nucleotides

The binding of GTP and FMN was evidenced in two sets of conditions (MES 10 mM pH 6.5 at 20°C, and HEPES 10 mM, NaCl 100 mM, pH 7.5 at 30°C) and involved hPEBP1 pocket as well. However, hPEBP1 shows a higher affinity for FMN than for GTP, in agreement with literature reports concerning bPEBP1 [28]. Moreover, a higher affinity was observed at pH 6.5/20°C than at pH 7.5/NaCl 100 mM/30°C for both GTP and FMN (Table 1). We carried out complementary experiments to differentiate the effect of the pH alone. Therefore, FMN was studied in HEPES 10 mM pH 7.5 at 20°C to compare with the binding study in MES 10 mM pH 6.5 at 20°C. Similar to the data at pH 6.5, the titration of FMN at pH 7.5 showed a majority of residues in slow exchange, but also residues in intermediate and fast exchange. Altogether, the perturbed residues defined the hPEBP1 pocket as the binding site of FMN at pH 7.5/20°C (data not shown). The measured affinity indicated no significant effect of the pH: K_D_ = 14±11 µM at pH 7.5/20°C (estimation from CSP data of 9 residues in fast exchange) versus K_D_ = 14±9 µM at pH 6.5/20°C (estimation from CSP data of 14 residues in fast exchange). Concerning the effect of salt alone, it is important to note that the presence of NaCl 100 mM induced no change on the HSQC spectrum of hPEBP1 indicating no conformational modification. Since the pH had no significant effect on FMN binding and no conformational change of the protein was induced by the presence of NaCl 100 mM, the decrease of nucleotides affinity in near physiological conditions was likely due to the temperature rise as a consequence of Van't Hoff law in chemical thermodynamics.

The interaction between hPEBP1 and GTP evidenced at pH 7.5/NaCl 100 mM/30°C was physiologically relevant despite the high concentrations of GTP used (from 0 to 6.4 mM to get saturation). Whereas the average concentration of GTP is 0.47 mM in mammalian cells and fluids [38], local concentration of GTP could be higher, particularly near the plasma membrane where receptors are coupled with heterotrimeric GTP-binding proteins. hPEBP1 is known to regulate G protein-coupled receptor signaling *in vivo* [39] and several studies have shown that hPEBP1 is associated with the G protein-coupled receptor kinase (GRK) [40]. In particular, hPEBP1 phosphorylated by PKC binds to GRK2 (G receptor kinase 2), inhibiting its activity and preventing receptor internalisation [14]. Thus, the GTP concentrations used in our experiments were certainly in the same order of magnitude as the GTP amounts encountered near the plasma membrane of living cells. Moreover, similar concentrations were used for GTP and FMN in order to compare the affinities obtained with both nucleotides.

**Figure 6:**
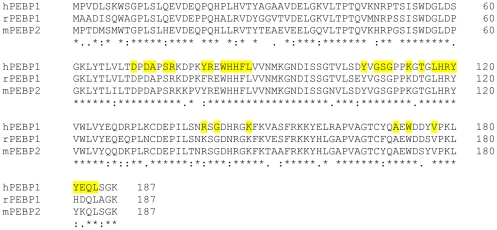
Multiple sequence alignment of human PEBP1 (hPEBP1, accession number P30086), rat PEBP1 (rPEBP1, accession number P31044) and mouse PEBP2 (mPEBP2, accession number Q8VIN1). The hPEBP1 residues defining the binding surface of GTP at pH 6.5 and 20°C are colored yellow.

The evidence of GTP binding to hPEBP1 in near physiological conditions contrasts with the NMR study of Shemon and co-workers (2010). These authors observed that GTP did not cause significant chemical shift perturbations for rPEBP1 at pH 7.4/NaCl 100 mM/30°C, even at a very high ligand concentration (130 mM GTP for 75 µM rPEBP1) [31]. Since similar conditions of pH, salt and temperature and identical NMR techniques were used in both studies, this result highlights the difference in binding behavior between the rat and the human PEBP1s in spite of an 83% sequence identity (Figure 6). Dadvar and co-workers (2009) have also evidenced different binding behaviors between PEBPs from two species [33]. In spite of an 84% sequence identity, the *in vitro* binding of an inhibitor of PDE5 was significantly more efficient with the mouse PEBP (mPEBP2) than with hPEBP1. Since PEBP has multiple isoforms in each species, and the number of isoforms is different from one species to another, it seems possible that the binding properties of a given PEBP are different from those of its counterpart in another species. Thus, the results obtained for one species cannot be generalized to the other.

### The binding of the Raf-1 peptide in tri-phosphorylated and non-phosphorylated forms

hPEBP1 pocket did bind the tri-phosphorylated Raf-1 peptide (Figure 3C), as previously shown by surface plasmon resonance [34], or for rPEBP [30], [35]. In particular, our data showed that residues A73 and S75 surrounding P74 as well as residue H86 were involved in the binding, supporting the study of Granovsky and co-workers (2009) that showed the effect of the mutations P74L and H86A in the pocket on the binding of rPEBP1 with Raf-1 kinase. Besides, although S153 was not perturbed itself, residues K150, V151, A152 immediately preceding S153 in α-helix H_1_ of hPEBP1 were affected by the tri-phosphorylated Raf-1 peptide binding. This could agree with the fact that rPEBP1 dissociates from Raf-1 upon phosphorylation by PKC on S153 (Figure 3C) [13], [14].

Raf-1 peptide binds more tightly when it is phosphorylated, as previously demonstrated by Park and co-workers [34]. As expected, the binding site of the non-phosphorylated Raf-1 peptide was centered on the conserved pocket (Figure 3D), involving residue G110 at the bottom of the pocket. However, most residues of the pocket were not involved in the binding. Indeed, residues in the vicinity of the pocket, rather than those within the pocket, were perturbed and hence, seemed to be required for interaction with Raf-1, as previously suggested by Shemon and co-workers (2009, 2010) [31], [32].

Since our data demonstrated differences between rat and human PEBP1s for GTP binding, we investigated the interaction with the locostatin precursor (S)-4-benzyl-2-oxazolidinone (Sigma #294640), for which the binding to rPEBP1 has been evidenced by NMR under near physiological conditions (Tris-HCl 50 mM pH 7.4, NaCl 100 mM, 30°C). The titration of the locostatin precursor with hPEBP1 (in HEPES 10 mM pH 7.5, NaCl 100 mM, 30°C) revealed 19 residues in fast exchange on the NMR time scale (data not shown). Analysis of the CSP values of these 19 residues provided a binding constant equal to 173±19 µM. The mapping of the perturbed residues on the X-ray structure of hPEBP1 shows that the locostatin precursor binds to the hPEBP1 pocket as previously shown for rPEBP1 under similar conditions (no K_D_ value was measured for rPEBP1) [32].

In the present study, we confirm the binding of the human PEBP1 to two nucleotides (GTP and FMN) and a Raf-1 peptide (in tri-phosphorylated and non-phosphorylated forms) in different conditions using NMR and mass spectrometry. All ligands bind to the same region centered on the conserved ligand-binding pocket of hPEBP1 previously identified by X-ray crystallography. Our work confirms that residues in the vicinity of the pocket rather than those within the pocket seem to be required for interaction with Raf-1 [31], [32]. The affinity constants K_D_ were estimated by NMR titration and/or native mass spectrometry. Although the affinities for GTP and FMN were lower at pH 7.5/NaCl 100 mM/30°C than at pH 6.5/20°C, both nucleotides clearly did bind under near physiological conditions. Since no interaction was shown between the rat PEBP and GTP by NMR in near physiological conditions [31], our study demonstrates the specific binding behavior of the human PEBP1 and highlights the importance of the studied species. In a therapeutic perspective, the choice to study human PEBP1 is a critical factor in drawing conclusions on human pathologies.

## Materials and Methods

Interaction of hPEBP1 was studied with four different ligands: two nucleotides, GTP and FMN, and a Raf-1 peptide of 19 amino acids in tri-phosphorylated and non-phosphorylated forms.

### Materials

Guanosine triphosphate (GTP), flavin mononucleotide (FMN), β-mercaptoethanol (BME), and ammonium bicarbonate (ABC) were purchased from Sigma (St. Louis, MO). Ammonium chloride ^15^N 98% (^15^NH_4_Cl) was purchased from Cortecnet (Voisins-Le-Bretonneux, France). Ammonium acetate (NH_4_OAc) was purchased from Merck (Darmstadt, Germany) and formic acid 90% (FA) from Fisher (Loughborough, UK). The Raf-1 peptide in tri-phosphorylated and non-phosphorylated forms was prepared by conventional solid-phase peptide synthesis using the Fmoc strategy. Fmoc-Ser(PO(OBzl)OH)-OH and Fmoc-Tyr(PO(OBzl)OH)-OH were used as phosphoderivatives. They were obtained by combining both a manual chain assembly method and an automated one with a ABI 433A synthesizer (Applied Biosystems). Details of the synthesis strategy will be described elsewhere. All solvents and buffers were prepared using 18 MΩ purified water (MilliQ reagent grade system, Millipore).

### Production and purification of ^15^N hPEBP1

^15^N hPEBP1 was produced according to the method described by Marley [41]. The cDNA coding the human PEBP1 has been inserted in pET31b plasmid [29]; *E. coli* BL21 DE3 cells were used to overexpress hPEBP1. The general protocol is as follows: 2 L of an E. coli BL21 (DE3, pET31b) overnight preculture were inoculated into 60 L of LB. Upon reaching OD_600_ ∼0.7, the cells were pelleted by centrifugation. The cell pellet was resuspended in 15 L of M9 medium with ^15^NH_4_Cl 1 g/L, ampicillin 50 µg mL^−1^, and then incubated to allow the recovery of growth and the clearance of unlabeled metabolites. After 1 h, protein expression was induced by addition of isopropyl-1-thio-β-galactoside (IPTG) to a final concentration of 1 mM. After a 2–3 h incubation period, the cells were harvested and frozen at −20°C.

The purification of hPEBP1 was performed according to a two-step procedure involving two different ion exchange chromatography columns. The frozen cell pellet was resuspended in water and loaded into a French Press cell disruptor. The cell lysate was centrifuged at 14,000 *g* for 20 min at 4°C. The clear supernatant was dialysed overnight against Tris 20 mM, EDTA 1 mM, BME 1 mM, pH 8.0. The dialysed cell lysate was loaded onto an anion exchange chromatography column (Q Sepharose Fast Flow, Amersham) and eluted with Tris 20 mM, BME 1 mM, pH 8.0. The fractions containing hPEBP1, identified with 18% SDS-PAGE, were gathered and dialysed overnight against NaAc 10 mM, BME 1 mM, pH 5.5. The dialysed sample was loaded onto a cation exchange chromatography column (Sp Sepharose High Performance, Amersham). hPEBP1 was eluted with a linear gradient 0–1 M NaCl. The fractions containing the protein were gathered and dialysed against MES 10 mM, BME 1 mM, pH 6.5. The protein solution was aliquoted and stored at 4°C. The final protein purity was assessed according to 18% SDS-PAGE gel and mass spectrometry.

### hPEBP1 and nucleotides purification for mass spectrometry analysis

Non-labeled recombinant hPEBP1 purified as previously described [29] was used for mass spectrometry analysis. To prevent Na^+^ adduct formation, the commercial GTP and FMN nucleotides used in native MS were desalted. For this purpose, a protocol derived from the RNA-desalting procedure of Limbach et al. (1995) [42] was set up [43].

### NMR measurements

The interactions between hPEBP1 100–270 µM and the four selected ligands were investigated by ^15^N-^1^H heteronuclear single quantum coherence (HSQC) NMR experiments with a sensitivity enhancement and gradient selected coherence. ^1^H, ^15^N HSQC spectra were recorded at 20 or 30°C on a Bruker 500 MHz or a Varian Inova 600 MHz spectrometer. Two experimental sets of conditions were tested: MES 10 mM pH 6.5 at 20°C, and HEPES 10 mM, NaCl 100 mM pH 7.5 at 30°C.

Although the backbone assignment is available for the human protein at pH 4/25°C at the BMRB (BMRB 16992) [44], we performed our own backbone amide assignment of free hPEBP1 at pH 6.5/25°C (BMRB 18204) using 3D TROSY-based HNCA, HN(CO)CA, HNCACB, HN(CO)CACB, HNCO and HN(CA)CO experiments [45]. ^1^H and ^15^N chemical shifts were assigned for 96.5% of non-prolines residues: all residues except Met1, Val3, Asp35, Gln45, Lys47 and Lys187 (total residues: 187; non-prolines residues: 172; assigned residues: 166/172). Measurements were performed on a Bruker Avance spectrometer 800 MHz equipped with a cryogenic ^1^H{^13^C/^15^N} triple-resonance probe.

### NMR titrations

In the simple case of protein-ligand interactions, the free and the bound states are observed during the titration. The interpretation of an NMR spectrum, such as an HSQC, depends on the rate of exchange between the bound and the free forms. Three different cases can be observed. If the complex rate of dissociation is very slow, two separate resonances are observed at the positions corresponding to the chemical shifts characteristic of the two states (free and bound). During the titration, the intensity of the free resonance decreases while the bound resonance one appears and goes up. This regime corresponds to slow chemical exchange on the NMR time scale. If the complex rate of dissociation is very fast, only a single resonance is observed, whose position is the average of the chemical shifts of the two states, weighted by their relative populations. In this case, Chemical Shift Perturbations (CSP) are observed, *i.e.* the chemical shift evolves as the ligand concentration increases. This regime corresponds to fast exchange on the NMR time scale, and is typical for weaker affinity complexes. In the intermediate chemical exchange case, in addition to CSP, complex changes will affect the line shape that results in the observation of very broad signals with low intensity.

In the fast exchange regime, CSP can be measured from ^15^N-HSQC spectra using the equation:
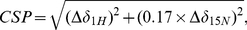
(1)with δ being the chemical shift in ppm [37].

A threshold value was estimated in order to determine significant CSP. In a first step, all the CSP are considered and the average (<CSP>) plus two times the standard deviation (σ) is calculated. Then, the highest CSP (CSP≥<CSP>+2σ) are removed from the data and new average and new standard deviation calculated. The operation is repeated until the convergence is reached. The final value <CSP>+2σ for the residues not significantly perturbed corresponds to the threshold.

Once the residues involved in the binding were selected, the experimental data were fitted with the quadratic equation 2 using SigmaPlot 9.0 in order to obtain the dissociation constant value (K_D_):
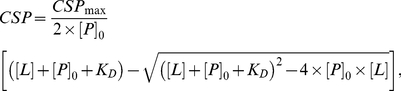
(2)where [P]_0_ and [L] are the total protein and ligand concentrations, respectively [46]. A K_D_ value was estimated for each residue involved in the binding, and then an average was calculated.

In the slow exchange regime, intensity ratios I/I_0_ can be calculated upon titration with I the peak intensity at a fixed concentration of ligand and I_0_ the initial peak intensity. A method similar to the one explained for CSP was used to discriminate the significant loss of intensity. The threshold corresponds to the average of the intensity ratios values (<I/I0>) minus two times the standard deviation (σ) for the residues not significantly perturbed.

Once the significant perturbations were discriminated, the perturbed residues were taken into account for the determination of the binding surface when (i) the perturbation reaches saturation upon titration, (ii) the residues are located at the surface, and (iii) define a contiguous surface patch [36].

In the case of the slow exchange regime, the binding constant can rarely be calculated because peak intensities are not measured with enough accuracy.

### Native mass spectrometry

All MS measurements were performed in an ESI-ion trap model Esquire HCT or Ultra HCT PTM Discovery (Bruker, Bremen, Germany), or in a maXis ESI*-*UHR*-*Qq*-*TOF (Bruker). Complexes were formed by incubating hPEBP1 with a range of ligand concentrations in ammonium bicarbonate 20 mM/formic acid buffer, pH 7.4 at 37°C or in ammonium acetate 20 mM, pH 6.6 at 20°C. After incubation, samples were treated with a Zeba micro gel filtration device with a 7 kDa cut-off (Thermo Scientific, Waltham, MA) prior to MS measurement, or analyzed directly in MS. The K_D_ was determined by measuring the bound protein fraction by native MS. Details of the development of the native MS method for K_D_ determination are described in the work of Jaquillard *et al.* (in press) [43].
